# Supplementary material for: Elongation roadblocks mediated by dCas9 across human genes modulate transcription and nascent RNA processing
Source: Nat Struct Mol Biol. 2023 Oct 2;30(10):1536–48. doi: 10.1038/s41594-023-01090-9 (PMC10584677; doi:10.1038/s41594-023-01090-9)
Supplement: Supplementary file 1 — Reporting Summary [file 41594_2023_1090_MOESM1_ESM.pdf]

nature portfolio

Corresponding author(s): Nicholas Proudfoot

Last updated by author(s): 16/05/23

Reporting Summary

Nature Portfolio wishes to improve the reproducibility of the work that we publish. This form provides structure for consistency and transparency in reporting. For further information on Nature Portfolio policies, see our Editorial Policies and the Editorial Policy Checklist.

Statistics

For all statistical analyses, confirm that the following items are present in the figure legend, table legend, main text, or Methods section.

n/a	Confirmed	
☐	☒	The exact sample size (n) for each experimental group/condition, given as a discrete number and unit of measurement
☐	☒	A statement on whether measurements were taken from distinct samples or whether the same sample was measured repeatedly
☐	☒	The statistical test(s) used AND whether they are one- or two-sided<br>*Only common tests should be described solely by name; describe more complex techniques in the Methods section.*
☒	☐	A description of all covariates tested
☒	☐	A description of any assumptions or corrections, such as tests of normality and adjustment for multiple comparisons
☐	☒	A full description of the statistical parameters including central tendency (e.g. means) or other basic estimates (e.g. regression coefficient) AND variation (e.g. standard deviation) or associated estimates of uncertainty (e.g. confidence intervals)
☐	☒	For null hypothesis testing, the test statistic (e.g. F, t, r) with confidence intervals, effect sizes, degrees of freedom and P value noted<br>*Give P values as exact values whenever suitable.*
☒	☐	For Bayesian analysis, information on the choice of priors and Markov chain Monte Carlo settings
☒	☐	For hierarchical and complex designs, identification of the appropriate level for tests and full reporting of outcomes
☒	☐	Estimates of effect sizes (e.g. Cohen's d, Pearson's r), indicating how they were calculated

Our web collection on statistics for biologists contains articles on many of the points above.

Software and code

Policy information about availability of computer code

Data collection

No software was used for data collection.

Data analysis

FastQC (v0.11.5) https://www.bioinformatics.babraham.ac.uk/projects/fastqc/
TrimGalore (v0.6.7) https://www.bioinformatics.babraham.ac.uk/projects/trim_galore/
Samtools (v1.11)12
http://samtools.sourceforge.net/ [12]
STAR (v2.7.0)13
https://github.com/alexdobin/STAR
Kallisto (v0.46.0)14
https://github.com/pachterlab/kallisto [14]
ggsashimi (v1.0.0)15
https://github.com/guigolab/ggsashimi [15]
pysam (v0.15.4)12
https://github.com/pysam-developers/pysam [12]
DESeq2 (v1.28.1)16
https://bioconductor.org/packages/release/bioc/html/DESeq2.html [16]
lfcShrink (1.20.0)17
http://bioconductor.org/packages/release/bioc/html/apeglm.html [17]
ggplot2 (v3.3.3)18
https://ggplot2.tidyverse.org
Bedtools (v2.30.0)19

https://bedtools.readthedocs.io/en/latest/content/bedtools-suite.html
bedGraphToBigWig http://genome.cse.ucsc.edu/index.html
UCSC genome browser http://genome.cse.ucsc.edu/index.html
R (v4.2.1) https://www.r-project.org
Python (v3.10.6) https://www.python.org

For manuscripts utilizing custom algorithms or software that are central to the research but not yet described in published literature, software must be made available to editors and reviewers. We strongly encourage code deposition in a community repository (e.g. GitHub). See the Nature Portfolio guidelines for submitting code & software for further information.

Data

Policy information about availability of data

All manuscripts must include a data availability statement. This statement should provide the following information, where applicable:

- Accession codes, unique identifiers, or web links for publicly available datasets
- A description of any restrictions on data availability
- For clinical datasets or third party data, please ensure that the statement adheres to our policy

All data needed to evaluate the conclusions in the paper are present in the paper. The NGS data were uploaded to the Genome Expression Omnibus (GSE228798 (https://www.ncbi.nlm.nih.gov/geo/query/acc.cgi?acc=GSE228798)
Note: the data are currently in private mode, accessible to view with reviewer token (see below). Once the manuscript is accepted, the data will go public.
To review GEO accession GSE228798:
Go to https://www.ncbi.nlm.nih.gov/geo/query/acc.cgi?acc=GSE228798
Enter token orapcqqqrvydtqn into the box

Human research participants

Policy information about studies involving human research participants and Sex and Gender in Research.

Reporting on sex and gender	N/A
Population characteristics	N/A
Recruitment	N/A
Ethics oversight	N/A

Note that full information on the approval of the study protocol must also be provided in the manuscript.

Field-specific reporting

Please select the one below that is the best fit for your research. If you are not sure, read the appropriate sections before making your selection.

☒ Life sciences ☐ Behavioural & social sciences ☐ Ecological, evolutionary & environmental sciences

For a reference copy of the document with all sections, see nature.com/documents/nr-reporting-summary-flat.pdf

Life sciences study design

All studies must disclose on these points even when the disclosure is negative.

Sample size	No statistical methods were used to pre-determine the sample size. Sample size was taken as number of biological replicates.
Data exclusions	No data were excluded.
Replication	All experiments and assays were confirmed with at least one replicate as described in the manuscript.
Randomization	All cell cultures were grown under identical conditions therefore randomization was not relevant for this study
Blinding	Blinding is not applicable for this study as it does not involve any subject assessment of the data that may influence validity of results.

Reporting for specific materials, systems and methods

We require information from authors about some types of materials, experimental systems and methods used in many studies. Here, indicate whether each material, system or method listed is relevant to your study. If you are not sure if a list item applies to your research, read the appropriate section before selecting a response.

Materials & experimental systems

n/a	Involved in the study
☐	☒ Antibodies
☐	☒ Eukaryotic cell lines
☒	☐ Palaeontology and archaeology
☒	☐ Animals and other organisms
☒	☐ Clinical data
☒	☐ Dual use research of concern

Methods

n/a	Involved in the study
☒	☐ ChIP-seq
☒	☐ Flow cytometry
☒	☐ MRI-based neuroimaging

Antibodies

Antibodies used

(Protein short name- Protein name - host animal - manufacturer - cat. no. - WB dilution and/or ChIP dilution)
Tub Tubulin Mouse Sigma T5168 1:10 000 –
FLAG anti-FLAG, M2, ChIP grade Mouse Sigma F3165 – 1 µg per 3 µg DNA
Pol II NTD ChIP grade Rpb1-NTD Rabbit CST #14958S 1:1 000 5 µl per 5 µg DNA
Pol II T4P ChIP grade Rpb1-CTD thr4P Rabbit CST #26319S – 5 µl per 5 µg DNA
HP1 γ ChIPAb+ validated HP1 gamma Mouse Sigma 17-646 – 5 µl per 3 µg DNA
H3 histone H3, ChIP grade Rabbit abcam ab1791 1:30 000 –
H3K9me2 H3K9me2 ChIP grade Mouse Abcam ab1220 1: 1 000 –
H3K9me3 H3K9me3, ChIP grade Rabbit abcam ab8898 1: 1 000 0.6 µg per 5 µg DNA
H3K9-ac H3K9 Acetyl, ChIP grade Rabbit abcam ab4441 1: 1 000 –
IRDye 800CW Goat anti-Mouse IgG Secondary Antibody Goat Licor P926-32210 1:10 000 –
IRDye 680RD Goat anti-Rabbit IgG Secondary Antibody Goat Licor P926-68071 1:10 000 –

Validation

All antibodies were obtained commercially and had been validated by the corresponding manufacturer

Eukaryotic cell lines

Policy information about cell lines and Sex and Gender in Research

Cell line source(s)

Please refer to the Methods section. HeLa cells (originally obtained from ATCC (CCL-2) and maintained in Proudfoot lab) and HCT116 XRN2-AID TIR1 (Gift from Steve West lab, (Eaton et al., 2018))

Authentication

not authenticated

Mycoplasma contamination

Cell lines tested negative for Mycoplasma contamination

Commonly misidentified lines
(See ICLAC register)

No misidentified cell lines were used in this study.
