# Supplementary material for: Elongation roadblocks mediated by dCas9 across human genes modulate transcription and nascent RNA processing
Source: Nat Struct Mol Biol. 2023 Oct 2;30(10):1536–48. doi: 10.1038/s41594-023-01090-9 (PMC10584677; doi:10.1038/s41594-023-01090-9)
Supplement: Supplementary file 5 — Unprocessed western blots and/or gels. [file 41594_2023_1090_MOESM5_ESM.pdf]

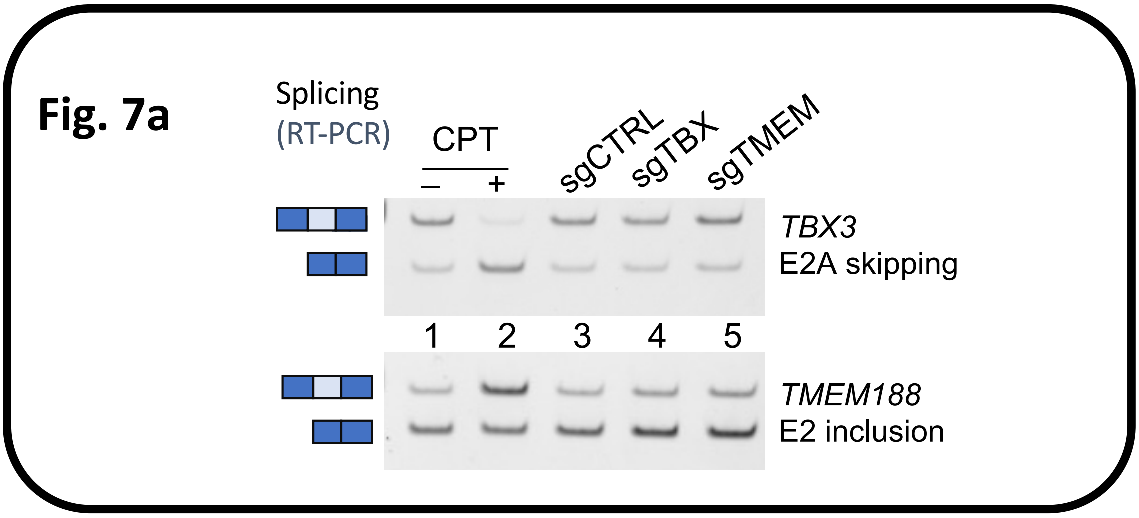


## TBX3

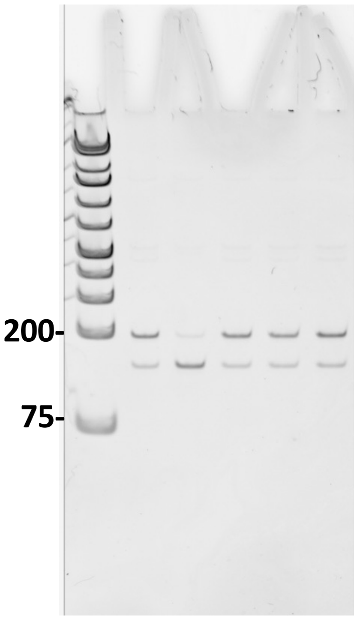


## TMEM188

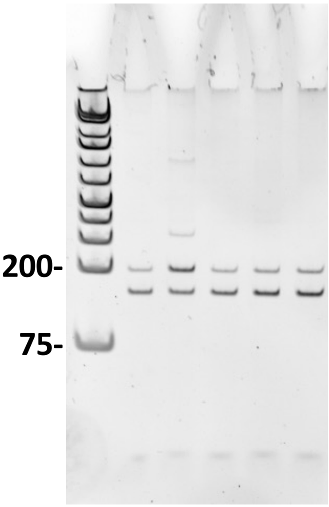

**Fig. 7c**

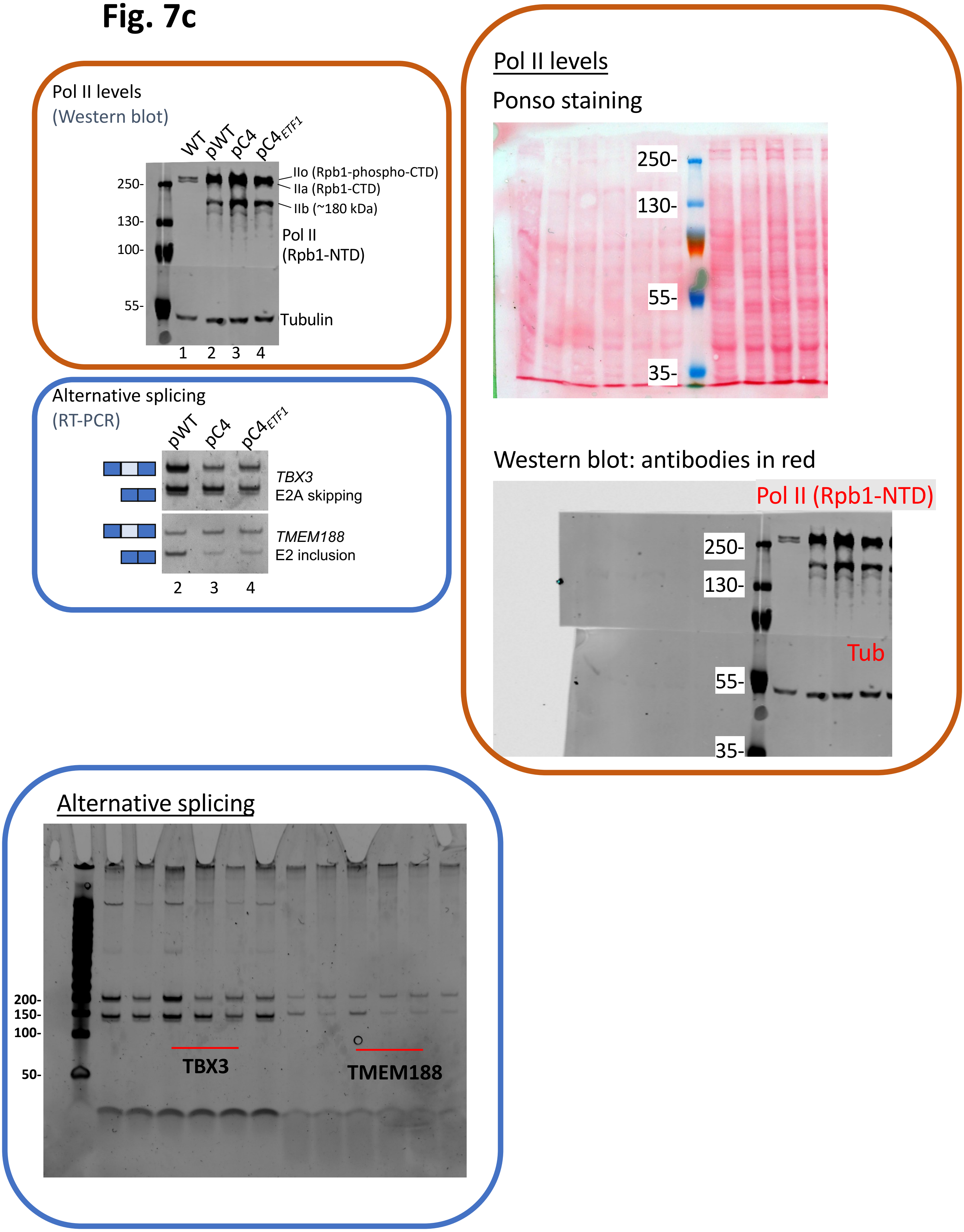

Pol II levels
(Western blot)
WT
pWT
pC4
pC4ETF1
250-
130-
100-
55-
IIo (Rpb1-phospho-CTD)
IIa (Rpb1-CTD)
IIb (~180 kDa)
Pol II
(Rpb1-NTD)
Tubulin
1 2 3 4
Alternative splicing
(RT-PCR)
pWT
pC4
pC4ETF1
TBX3
E2A skipping
TMEM188
E2 inclusion
2 3 4
Pol II levels
Ponso staining
250-
130-
55-
35-
Western blot: antibodies in red
Pol II (Rpb1-NTD)
250-
130-
Tub
55-
35-
Alternative splicing
200-
150-
100-
50-
TBX3
TMEM188
