# Supplementary material for: Elongation roadblocks mediated by dCas9 across human genes modulate transcription and nascent RNA processing
Source: Nat Struct Mol Biol. 2023 Oct 2;30(10):1536–48. doi: 10.1038/s41594-023-01090-9 (PMC10584677; doi:10.1038/s41594-023-01090-9)

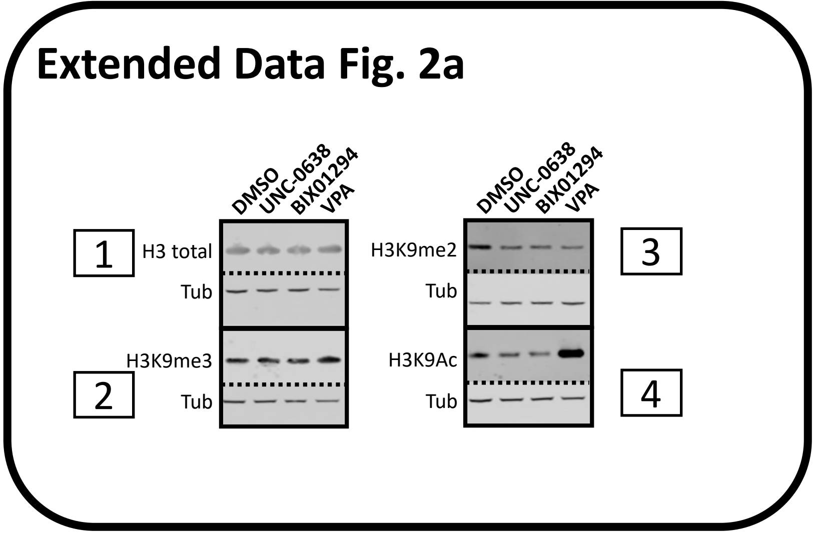

Ponso staining:

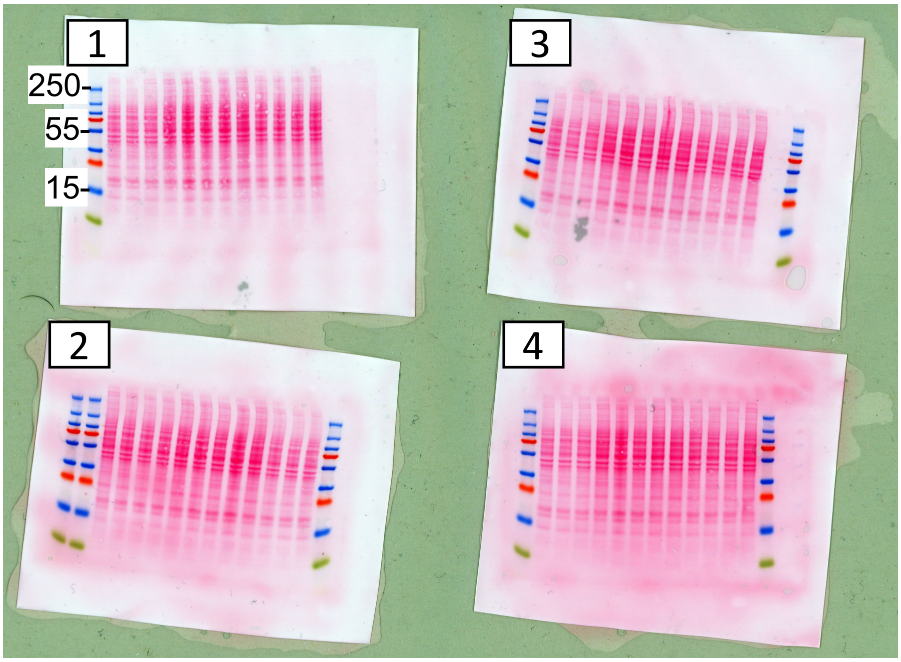

Western blot: red lines show lanes used in figure, red font– the antibody used.

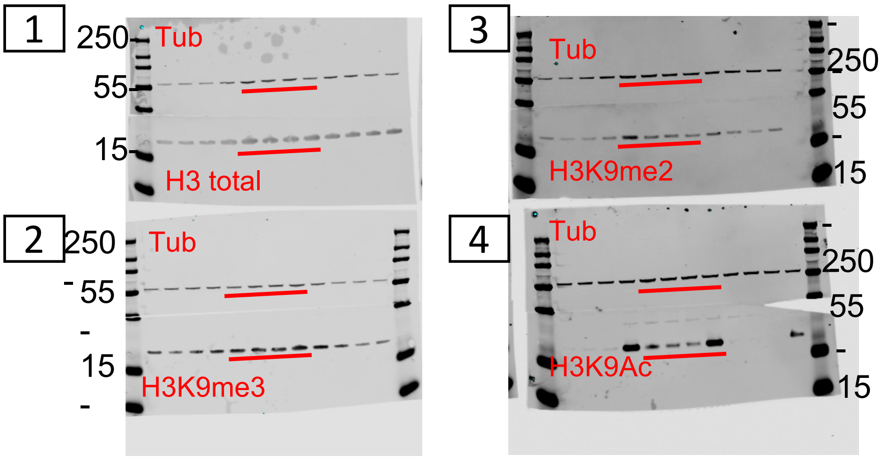

Supplement: Supplementary file 6 — Unprocessed western blots and/or gels. [file 41594_2023_1090_MOESM6_ESM.pdf]
